# Channel Aperture Characteristics of Carbonate Apatite Honeycomb Scaffolds Affect Ingrowths of Bone and Fibrous Tissues in Vertical Bone Augmentation

**DOI:** 10.3390/bioengineering9110627

**Published:** 2022-10-31

**Authors:** Koichiro Hayashi, Ryo Kishida, Akira Tsuchiya, Kunio Ishikawa

**Affiliations:** Department of Biomaterials, Faculty of Dental Science, Kyushu University, Fukuoka 812-8582, Japan

**Keywords:** scaffold, honeycomb, apatite, bone, tissue engineering

## Abstract

Synthetic scaffolds with the ability to prevent fibrous tissue penetration and promote bone augmentation may realize guided bone regeneration without the use of a barrier membrane for dental implantation. Here, we fabricated two types of honeycomb scaffolds of carbonate apatite, a bone mineral analog, whose channel apertures were square (HC-S) and rectangular (HC-R). The side lengths of the HC-Ss and HC-Rs were 265.8 ± 8.9; 817.7 ± 2.4 and 267.1 ± 5.2 μm, respectively. We placed cylindrical HC-Ss and HC-Rs on the rabbit calvaria. At 4 weeks post-implantation, the HC-Ss prevented fibrous tissue penetration from the top face via the channels, which allowed the new bone to reach the top of the scaffold from the bottom face or the calvarium. In contrast, in the HC-Rs, fibrous tissues filled the channels in the top region. At 12 weeks post-implantation, the HC-Ss were partially replaced with new bone. In the top region of the HC-Rs, although new bone had formed, fibrous tissue remained. According to the findings here and in our previous study, the longer side length rather than the shorter side length of a rectangular scaffold channel aperture is the dominant factor that affects fibrous tissue penetration and new bone augmentation. Furthermore, even though channel aperture areas are similar, bone and fibrous tissue ingrowths are different when the aperture shapes are different.

## 1. Introduction

Dental implantation is becoming increasingly important with the aging of the global population [1,2,3,4]. When the volume of the alveolar bone is insufficient for implant placement, bone augmentation is required [1,2,3,4]. Notably, vertical bone augmentation is the most difficult treatment among bone augmentations for various bone loss configurations [5,6,7,8].

Guided bone regeneration (GBR) is a common technique for bone augmentation [5,6,7,8,9,10,11,12,13,14,15,16,17,18]. In GBR, a barrier membrane is placed between fibrous tissues and the bone defect to prevent the undesirous incursions of fibrous tissues into the bone defect, thereby allowing the regeneration of bone. However, handling the barrier membrane is difficult, and therefore failures, such as fibrous tissue penetration and barrier membrane exposure, and complications, such as infection, frequently occur [5,6,7,8,9,10,11,12,13,14,15,16,17,18]. Furthermore, when non-resorbable barrier membranes are used, secondary surgery for removing the barrier membrane is required [5,8,16,17]. In contrast, resorbable barrier membranes are resorbed faster than bone formation, providing insufficient bone volume and mechanical strength [11,18]. Considering the above problems in the use of barrier membranes, GBR with only bone substitute materials and without a barrier membrane, namely membrane-free GBR, is considered an ideal treatment.

As bone substitute materials, autologous bone is commonly used [14,15]. However, autogenous bone grafts have several drawbacks, such as donor site morbidity, soft tissue and nerve injuries, post-surgery pain, limited harvest quantity, and increased surgical operating time [19,20,21,22,23]. Therefore, the development of alternative scaffolds to autologous bone is necessary [24,25,26,27,28,29,30,31].

So far, we have developed several types of porous calcium phosphate scaffolds and demonstrated the effects of scaffold composition [32,33], shape [34], and pore structure at the macro- [35,36,37,38,39,40,41,42,43,44,45,46], micro- [44,45,46,47], and nanoscale [48] on osteogenesis and angiogenesis. Regarding scaffold composition, carbonate apatite (CAp) bone mineral was resorbed by osteoclasts and showed superior osteoconductivity to hydroxyapatite (HAp) and β-tricalcium phosphate (β-TCP) [32,33]. As for pore structure, uniaxial through-channels, such as square channels in a honeycomb (HC) filter for exhaust gas purification, promoted ingrowths of bone and blood vessels [32,33,34,35,36,37,38,39,40,41,42,43,44,45,46,47,48]. HC channels that uniaxially penetrate the scaffold from the bottom surface to the top are considered to prevent fibrous tissue penetration from the scaffold side surface, although scaffolds with three-dimensional porous structures may not. Additionally, in vertical bone augmentation, the contact region of the scaffold with the bone is limited to the scaffold’s bottom surface. From this perspective as well, HC seems to be a suitable structure for vertical bone augmentation. Furthermore, the side length of the square channel aperture affected the penetration of fibrous tissues into the scaffold [41]: channels with aperture side lengths of 200–300 μm prevented fibrous tissue penetration, whereas channels with aperture side lengths > 400 μm could not [41]. These results indicated that controlling the characteristics of the channel aperture may achieve membrane-free GBR.

Although we previously demonstrated the effects of square channels with four equal sides on bone formation and the prevention of fibrous tissue penetration, we did not investigate the effects of rectangular channels with adjacent sides of different lengths. Therefore, in rectangular channels where one side length is effective for preventing fibrous tissue penetration, i.e., 200–300 μm, and the other side length is not effective, i.e., >400 μm, it is unclear which will be dominant—the shorter or longer length. Here, we explore the answer to this question. Furthermore, we discuss the channel aperture parameters affecting bone augmentation and fibrous tissue penetration.

## 2. Materials and Methods

### 2.1. Fabrication of HC Scaffolds with Square and Rectangular Channels

HC scaffolds with square channels (HC-S) were fabricated by the method described in our previous paper [35,41]. In brief, HC green bodies were prepared by the extrusion molding of a mixture of CaCO_3_ (Sakai Chemical Industry Co., Ltd., Osaka, Japan) and methylcellulose-based binder (Matsumoto Yushi-Seiyaku, Osaka, Japan) using an extruder (V-30 [II]; Universe, Saga, Japan) equipped with a die with square slits of 300 μm × 300 μm. The HC green bodies were subjected to debinding by heat treatment at 600 °C for 24 h, resulting in the formation of CaCO_3_ HC materials. Then, the CaCO_3_ HC materials were phosphatized by immersion in a 1 mol/L Na_2_HPO_4_ aqueous solution at 80 °C for 7 d. As a result, the composition of HC materials changed from CaCO_3_ to CAp. Finally, the obtained CAp HC materials were washed five times with distilled water and used as HC-Ss.

HC scaffolds with rectangular channels (HC-R) were also fabricated by extrusion molding, debinding, and phosphatization. Only the design of the die used in the extrusion molding was different from that of HC-S (rectangular slits of 300 μm × 900 μm). The debinding and phosphatization procedures for fabricating HC-Rs were the same as those for HC-Ss.

For animal experiments, HC-Rs and HC-S were shaped into cylinders (6 mm in diameter and 4 mm in height) using a three-dimensional (3D) milling machine (SRM-20; Roland DG, Shizuoka, Japan). The shaped scaffolds were subjected to dry-heat sterilization at 170 °C for 3 h.

### 2.2. Characterization of the HC Scaffold Structures and Compositions

The structures of HC-S and HC-R were characterized using micro-computerized tomography (μ-CT; SkyScan, Bruker, MA, USA) and scanning electron microscopy (SEM; S3400N; Hitachi High-Technologies, Tokyo, Japan).

The crystal phases of HC-S and HC-R were analyzed using X-ray diffraction (XRD; D8 Advance, Bruker AXS GmbH, Karlsruhe, Germany). Commercial CAp (Cytrans; GC, Tokyo, Japan) was used as the reference.

The functional groups in HC-S and HC-R were analyzed using Fourier transform infrared (FT-IR) spectroscopy (FT/IR-6200; JASCO, Inc., Tokyo, Japan). HAp powder was used as the reference.

### 2.3. Approval for Animal Experiments and Animal Rearing

The animal experiments were approved by the Animal Care and Use Committee of Kyushu University (No. A22-086-0). Japanese white rabbits (18 weeks old, male, body weight 3.0–3.5 kg) were purchased from Japan SLC (Shizuoka, Japan). The rabbits were housed in the Center of Biomedical Research, Research Center for Human Disease Modeling, Graduate School of Medical Sciences, Kyushu University. The rabbits were maintained on a standard diet (LRC4; Oriental Yeast, Tokyo, Japan) and were provided with water ad libitum.

### 2.4. Surgical Procedure

The rabbits were anesthetized via intramuscular injection of a mixture of xylazine (5.0 mg/kg; Elanco, IN, USA) and ketamine (30 mg/kg; Daiichi-Sankyo, Tokyo, Japan). The fur of the rabbit parietal region was shaved, disinfected with 10% *w*/*v* povidone-iodine (Meiji Seika Pharma, Tokyo, Japan), and subjected to subcutaneous injection of local anesthesia (Lidocain; Dentsply Sirona, NC, USA). The skin of the parietal region was incised to expose the calvarium. Then, the periosteum was separated from the bone. Two scaffolds (HC-R and HC-S) were randomly placed on the calvarium in each animal. The fasciae were sutured on the top surfaces of the scaffolds. Finally, the incised skin was sutured, and an antibacterial agent (gentamicin; Takata Pharmaceutical, Saitama, Japan) was peritoneally injected. The rabbits were allowed unrestrained movement in their cages after they recovered from the anesthesia.

### 2.5. Histological Analysis

The rabbits were euthanized in batches at 4 and 12 weeks after implantation of the HC-S and HC-R. The calvarium was harvested (*n* = 5 per group) and fixed with 10% formalin solution (Fujifilm Wako, Osaka, Japan) at 4 °C for 48 h. The specimens were dehydrated using a graded series of alcohol solutions and embedded in polymethyl methacrylate histological resin (Technovit 9100; Heraeus Kulzer, Wehrheim, Germany). All specimens were cut into longitudinal sections throughout the cylindrical scaffold, and hematoxylin and eosin (H&E)-staining was performed. The histological analysis of H&E-stained tissue sections was performed using a biological fluorescence microscope (BZ-X; Keyence Corporation, Osaka, Japan) with digital analysis software (BZ-X; Keyence Corporation). The area percentage of newly formed bone in HC-S and HC-R was calculated as new bone area per the whole area of the scaffold, including channels and struts. Furthermore, area percentages of new bone and fibrous tissues in each channel were calculated.

### 2.6. Statistical Analysis

All data are presented as the mean ± standard deviation. Results with *p* < 0.05 were considered statistically significant. Comparisons between groups were performed using Student’s *t*-test.

## 3. Results

### 3.1. Characterization of Scaffold Structures and Composition

**Figure 1:**
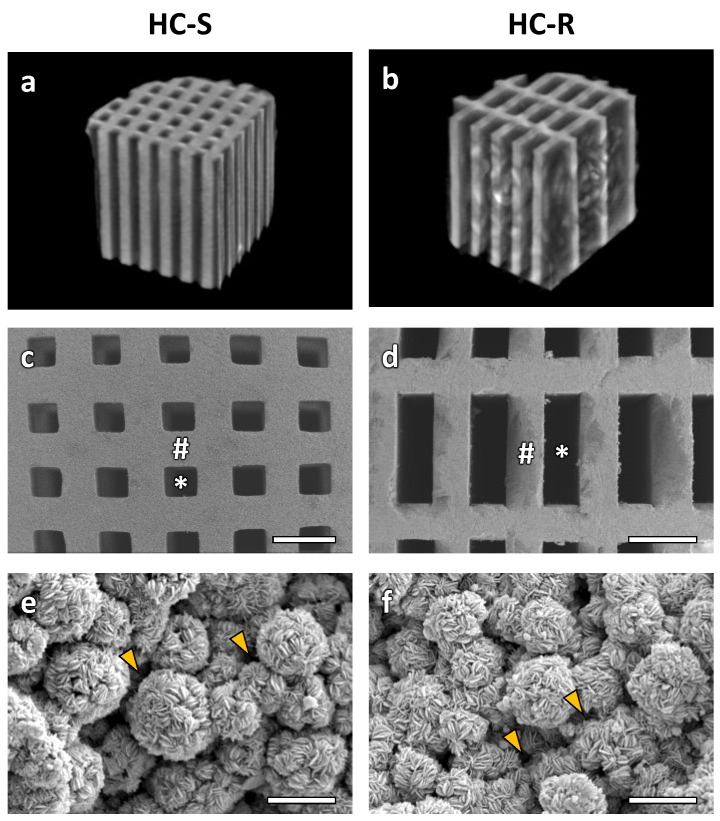
Micro-computerized tomography (μ-CT) cross-section images of honeycomb scaffolds with square channels (HC-S) (**a**) and honeycomb scaffolds with rectangle channels (HC-R) (**b**). Scanning electron microscopy (SEM) images of HC-S (**c**) and HC-R (**d**). Scale bars, 500 μm. # and * indicate struts and channels, respectively. Higher magnification SEM images of the struts in HC-S (**e**) and HC-R (**f**). Scale bars, 5 μm. Yellow arrowheads indicate micropores between carbonate apatite (CAp) spheres.

The μ-CT cross-section images showed that HC-S (Figure 1a) and HC-R (Figure 1b) possessed square and rectangular channels, respectively. These channels uniaxially penetrated the scaffolds. The SEM image of HC-S (Figure 1c) showed that the side length of the square channel aperture was 265.8 ± 8.9 μm. In HC-R (Figure 1d), the longer and shorter side lengths of the rectangular channel aperture were 817.7 ± 2.4 and 267.1 ± 5.2 μm, respectively. Thus, the shorter side length of the HC-R channels was almost equal to the side length of the HC-S channels, whereas the longer side of the HC-R channels was three times as long. The strut lengths of the HC-Ss (Figure 1c) and HC-Rs (Figure 1d) were 282.7 ± 9.4 and 278.3 ± 6.4 μm, respectively; thus, the HC-S struts were almost equal in size to the HC-R struts. Furthermore, in the struts of both HC-Ss (Figure 1e) and HC-Rs (Figure 1f), micropores were present between the spherical aggregates consisting of scale-like crystals. Many researchers previously demonstrated that micropores improved osteogenesis [47,48,49,50,51,52,53,54,55,56,57]. Therefore, the micropores in the HC-Ss and HC-Rs may also contribute to bone regeneration.

**Figure 2:**
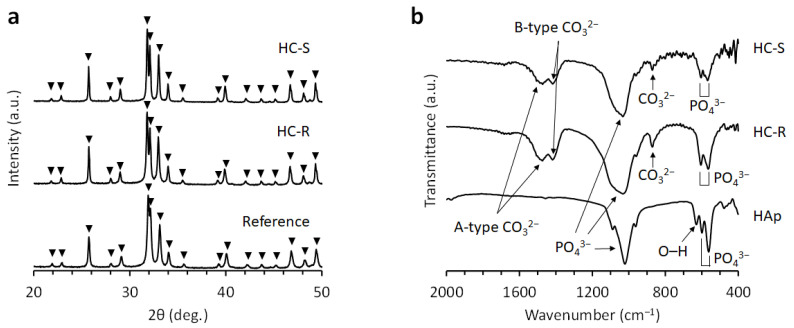
(**a**) X-ray diffraction patterns of honeycomb scaffolds with square channels (HC-S) and honeycomb scaffolds with rectangle channels (HC-R). Commercial carbonate apatite was used as a reference. (**b**) Fourier transform infrared (FT-IR) spectra of HC-S, HC-R, and hydroxyapatite (HAp).

The X-ray diffraction patterns of HC-S and HC-R corresponded to those of the CAp reference (Figure 2a). The FTIR spectra of HC-S and HC-R confirmed the absorption bands identified as PO_4_^3−^ group in apatite crystal (Figure 2b) [58,59]. Although the band identified as the OH^−^ group was present in the spectrum of HAp [58,59], it was absent in the spectra of HC-S and HC-R. Furthermore, A-type CO_3_^2−^ that substituted OH^−^ and B-type CO_3_^2−^ that substituted PO_4_^3−^ were confirmed in the spectra of HC-S and HC-R [58,59]. In contrast, no CO_3_^2−^bands were detected in the HAp spectrum. These results demonstrated that HC-S and HC-R were composed of CAp and that CO_3_^2−^ had replaced both PO_4_^3−^ and OH^−^ groups in HAp.

### 3.2. In Vivo Evaluations of Bone Formation and Fibrous Tissue Invasion

**Figure 3:**
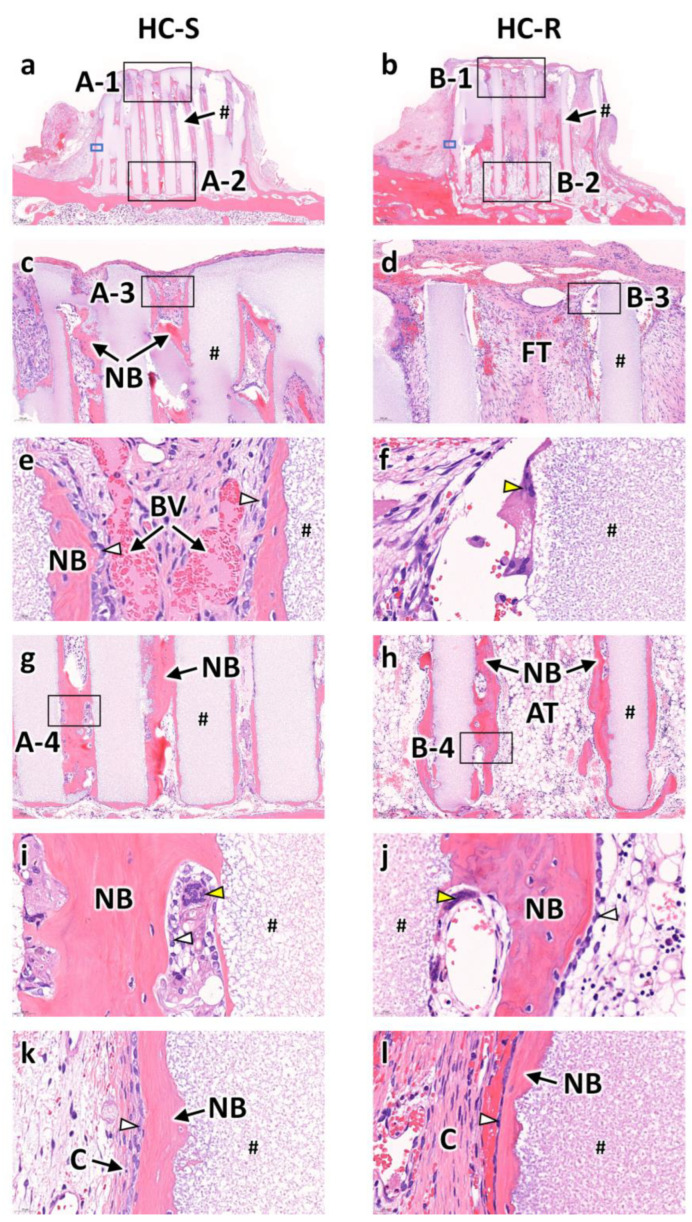
Hematoxylin and eosin (H&E)-stained sections at 4 weeks after implantation of honeycomb scaffolds with square channels (HC-S) (**a**) and honeycomb scaffolds with rectangle channels (HC-R) (**b**) on the rabbit calvaria. High magnification images of regions A-1 (**c**), B-1 (**d**), A-3 (**e**), B-3 (**f**), A-2 (**g**), B-2 (**h**), A-4 (**i**), and B-4 (**j**). High magnification images of regions enclosed in blue squares in Figure 3a (**a**) and Figure 3b (**b**). #, NB, FT, BV, AT, and C indicate scaffold strut, new bone, fibrous tissue, blood vessel, adipose tissue, and collagen fiber, respectively. White and yellow arrowheads indicate osteoblasts and osteoclasts, respectively.

Bone formation and fibrous tissue invasion were visualized by analyzing the H&E stained sections of HC-S and HC-R. At 4 weeks after implantation, in both the HC-Ss (Figure 3a) and HC-Rs (Figure 3b), tissues penetrated the scaffold channels. In the HC-Ss, new bone formed on the struts and reached the top region of the scaffold (Figure 3c). In contrast, in HC-R, fibrous tissues filled the channels in the top region (Figure 3d). High-magnification images of the top regions demonstrated that osteoblasts lined the new bone formed on the struts and blood vessels formed in the central regions of channels (Figure 3e). In the HC-Rs, multinuclear cells were present on the struts (Figure 3f). In the bottom regions of the scaffolds, new bone predominated in the HC-Ss (Figure 3g), whereas adipose tissue rather than bone was dominant in the HC-Rs (Figure 3h). High-magnification images of the bottom regions showed that osteoblasts were present on the new bone, and multinuclear cells were detected near the struts in both the HC-Ss (Figure 3i) and HC-Rs (Figure 3j). New bone also formed on the outer surfaces of HC-Ss and HC-Rs, and collagen tissues were present around the outside of the new bone (Figure 3k,l).

**Figure 4:**
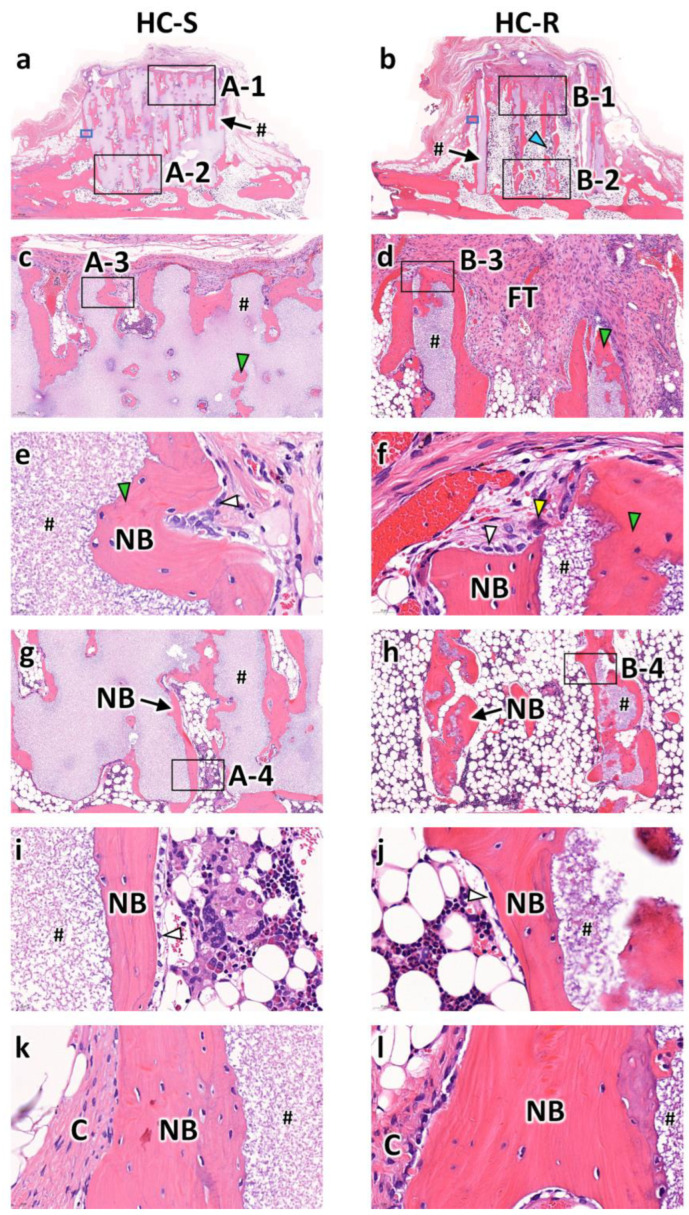
Hematoxylin and eosin (H&E)-stained sections at 12 weeks after implantation of honeycomb scaffolds with square channels (HC-S) (**a**) and honeycomb scaffolds with rectangle channels (HC-R) (**b**) on the rabbit calvaria. High magnification images of regions A-1 (**c**), B-1 (**d**), A-3 (**e**), B-3 (**f**), A-2 (**g**), B-2 (**h**), A-4 (**i**), and B-4 (**j**). High magnification images of regions enclosed in blue squares in Figure 4a (**a**) and Figure 4b (**b**). #, NB, FT, and C indicate scaffold struts, new bones, fibrous tissue, and collagen fiber, respectively. White, yellow, blue, and green arrowheads indicate osteoblasts, osteoclasts, resorbed part of the strut, and new bone formed in the resorbed strut part, respectively.

At 12 weeks post-implantation, in both the HC-Ss (Figure 4a) and HC-Rs (Figure 4b), the struts were partly resorbed. In the top region of the HC-Ss (Figure 4c), the area of new bone increased from that at 4 weeks post-implantation. In the top region of the HC-Rs (Figure 4d), bone was newly formed, although it was not observed at 4 weeks post-implantation. Nevertheless, fibrous tissues remained. High-magnification images of the top regions showed that the resorbed regions of the struts were replaced by new bone in both the HC-Ss (Figure 4e) and HC-Rs (Figure 4f). Likewise, in the bottom regions of the HC-Ss (Figure 4g) and HC-Rs (Figure 4h), the struts were partly replaced by new bone. Furthermore, osteoblasts were found on the surface of the new bone (Figure 4i,j). Furthermore, collagen tissues were present around the outside of the new bone formed on the HC-S and HC-R outer surfaces (Figure 4k,l).

**Figure 5:**
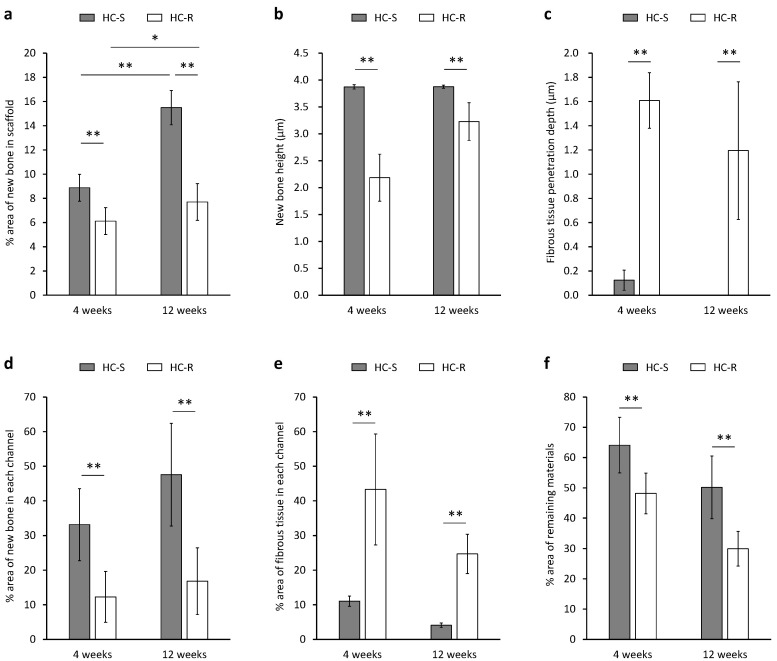
Area percentage (**a**) and height (**b**) of bone newly formed in honeycomb scaffolds with square channels (HC-S) and honeycomb scaffolds with rectangle channels (HC-R). The penetration depth of fibrous tissue from the scaffold top face (**c**). Area percentages of new bone (**d**) and fibrous tissues (**e**) in each channel of HC-S and HC-R. Area percentages of remaining materials in HC-S and HC-R (**f**). * *p* < 0.05 and ** *p* < 0.01. *n* = 5 per group.

New bone and fibrous tissues formed in the scaffolds were analyzed using the histological sections (Figure 5). The area percentage of new bone formed in the whole of HC-Ss (8.9 ± 1.1%) was significantly higher than that in the whole of HC-Rs (6.1 ± 1.1%) at 4 weeks after implantation (*p* = 0.002, Figure 5a). New bone percentages in the whole of HC-Ss and HC-Rs significantly increased during the 8 weeks between 4 weeks and 12 weeks post-implantation (*p* = 5.2 × 10^–5^ and 0.04, respectively). Nevertheless, the increase during the 8 weeks in the HC-Ss (75%) was higher than that in the HC-Rs (26%). Consequently, the new bone percentage in HC-S (15.5 ± 1.4%) was two-fold higher than that in HC-R (7.7 ± 1.5%) at 12 weeks post-implantation. The heights of bone newly formed in the HC-Ss and HC-Rs were 3.9 ± 0.1 and 2.2 ± 0.4 mm at 4 weeks post-implantation and 3.9 ± 0.1 and 3.3 ± 0.4 mm at 12 weeks post-implantation, respectively (Figure 5b). Thus, the new bone had already reached the top of the HC-Ss at 4 weeks, and the new bone height remained at 12 weeks. In contrast, in the HC-Rs, the new bone height was merely half that of the scaffold height at 4 weeks and was significantly inferior to that in HC-S even at 12 weeks (*p* = 2.9 × 10^–4^). The penetration depth of fibrous tissues in HC-S and HC-R were 0.1 ± 0.1 and 1.6 ± 0.2 mm at 4 weeks post-implantation and 0.0 ± 0.0 and 1.2 ± 0.6 mm at 12 weeks post-implantation, respectively (Figure 5c). The percentages of new bone in each channel of HC-Ss and HC-Rs were 33.1 ± 10.4 and 12.3 ± 7.4% at 4 weeks post-implantation and 47.6 ± 14.9 and 16.8 ± 9.7% at 12 weeks post-implantation, respectively (Figure 5d). Area percentages of fibrous tissues in each channel of HC-Ss and HC-Rs were 11.0 ± 1.5 and 43.3 ± 16.0% at 4 weeks post-implantation and 4.1 ± 0.7 and 24.7 ± 5.7% at 12 weeks post-implantation, respectively (Figure 5e). Thus, the dominant tissue in the channels was bone for HC-Ss, and fibrous tissues for HC-Rs. The above results demonstrate that HC-S could prevent the penetration of fibrous tissues, whereas HC-R could not. Furthermore, most of the fibrous tissues that penetrated HC-R remained at 12 weeks. These findings demonstrate that once fibrous tissue penetrates the scaffold, it is difficult for them to be replaced by bone, and therefore, the prevention of fibrous tissue penetration is important. The percentages of remaining materials for HC-Ss and HC-Rs were 64.1 ± 9.2 and 48.1 ± 6.7% at 4 weeks post-implantation and 50.1 ± 10.3 and 29.9 ± 5.7% at 12 weeks post-implantation, respectively (Figure 5f).

## 4. Discussion

We previously compared the bone formation and fibrous tissue penetration capability of CAp HC scaffolds with square channels of side lengths 230, 460, and 630 μm in the same animal experimental system as this study [41]. The scaffolds were named HC230, HC460, and HC630, respectively. In the previous study, HC230 could prevent the penetration of fibrous tissues into the scaffold, whereas HC460 and HC630 could not. In the present study, the side length of the square channels in the HC-Ss was ~265 μm, which is almost equal to that of HC230. Thus, the finding that HC-Ss can prevent the penetration of fibrous tissues is in accordance with our previous findings. The shorter and longer side lengths of the rectangular channel in HC-R were ~265 and ~820 μm, respectively. Thus, the shorter side length coincides with the side length that is effective for preventing fibrous tissue penetration, and the longer side length does not. Considering that HC-R could not prevent fibrous tissue penetration, it can be concluded that fibrous tissue penetration is influenced by the longer side length rather than the shorter side length.

**Figure 6:**
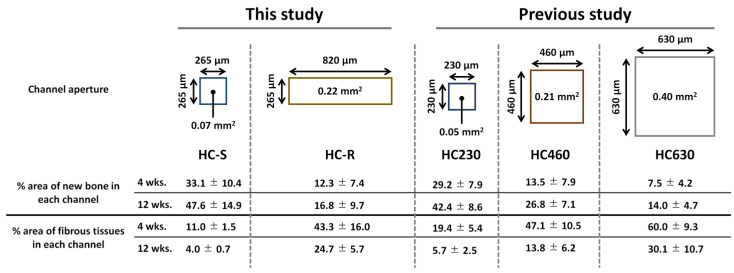
Channel aperture characteristics and the percent areas of new bone and fibrous tissues in each channel of honeycomb scaffolds with square channels (HC-S), with rectangle channels (HC-R), and with square channels of side lengths 230 (HC230), 460 (HC460), and 630 μm (HC630) [41].

The channel aperture areas of HC-S, HC-R, HC230, HC460, and HC630 were 0.07, 0.22, 0.05, 0.21, and 0.40 mm^2^, respectively (Figure 6) [41]. Thus, the HC-S and HC-R possessed aperture areas similar to those of HC230 and HC460, respectively. In our previous study, the percent areas of new bone in each channel for HC230, HC460, and HC630 were as follows: 29.2 ± 7.9%, 13.5 ± 7.9%, and 7.5 ± 4.2% at 4 weeks post-implantation and 42.4 ± 8.6%, 26.8 ± 7.1%, and 14.0 ± 4.7% at 12 weeks post-implantation, respectively (Figure 6) [41]. Furthermore, the percent area of fibrous tissues in each channel for HC230, HC460, and HC630 were as follows: 19.4 ± 75.4%, 47.1 ± 10.5%, and 60.0 ± 9.3% at 4 weeks post-implantation and 5.7 ± 2.5%, 13.8 ± 6.2%, and 30.1 ± 10.7% at 12 weeks post-implantation, respectively (Figure 6) [41]. Thus, at 4 and 12 weeks post-implantation, HC-S showed percent areas of new bone and fibrous tissues in each channel similar to those of HC230. These results demonstrate that when the channel aperture shape is square, scaffolds with similar channel aperture areas show similar behaviors of bone and fibrous tissue ingrowths. In contrast, although the percent areas of new bone and fibrous tissues in each channel for HC-R at 4 weeks post-implantation was similar to that for HC460, the percent area at 12 weeks post-implantation was closer to that for HC630 rather than HC460. Thus, when the channel aperture shape is rectangular, at 12 weeks post-implantation, the longer length of the channel aperture rather than the aperture area has greater effects on bone and fibrous tissue ingrowths. Therefore, we should design the pore structure of scaffolds with consideration for the aperture shape and area.

Previously, we demonstrated the relationship between the strut thickness of the HC scaffold and the resorption rate [35]. HC scaffolds of strut thicknesses 100, 200, and 300 μm named HC100, HC200, and HC300, respectively, were studied. The scaffolds possessed channels with square aperture side lengths of 230–260 μm. The remaining material percentages of HC100, HC200, and HC300 were 26.7 ± 6.4%, 58.0 ± 4.4%, and 73.7 ± 2.6% at 4 weeks post-implantation and 18.7 ± 3.0%, 39.2 ± 8.5%, and 68.1 ± 0.7% at 12 weeks post-implantation, respectively. In this study, the strut thickness of HC-S was 282.7 ± 9.4 μm, which is intermediate between that of HC200 and HC300. The remaining material percentages of HC-S at 4 and 12 weeks were 64.1 ± 9.2 and 50.1 ± 10.3%, respectively, which are also intermediate between those of HC200 and HC300. Thus, the results on material resorption in this study are consistent with those in our previous study. Furthermore, the histological sections at 12 weeks post-implantation in this study show that the resorbed regions in HC-S were filled with new bone, which may contribute to maintaining the continuity of the struts. In contrast, in HC-R, the resorbed regions were not completely replaced with new bone; consequently, HC-Rs lost strut continuity. Thus, the resorption speed of HC-S seems to coincide with new bone formation, whereas that of HC-R seems to be faster than new bone formation.

At present, clinical treatment involves implantation of autologous bone block grafts (BGs) or synthetic granular grafts (GGs), such as HAp and β-TCP, for bone augmentation. Autologous bone BGs can be configured to correspond with the bone defect configuration. However, the implantation of autologous bone BGs perforce causes donor site morbidity and frequently produces faster or slower resorption of the BGs than expected due to individual differences and BG conditions [14]. Synthetic GGs contribute to resolving these problems of autologous bone implantation. However, when synthetic GGs are implanted, a titanium mesh is often used to hold the GGs and generate desired configuration and volume of bone. Unfortunately, titanium mesh exposure occurs in 24% of patients, resulting in partial or complete graft loss and dental implant failure [60]. Furthermore, when titanium mesh is used, secondary surgery to remove it is necessary [60]. Synthetic BGs that can be configured to correspond with bone defect configuration can eliminate the use of titanium mesh, alleviate donor site morbidity, and produce expected outcomes. Thus, synthetic BGs may resolve the disadvantages of autologous bone BGs and synthetic GGs. Nevertheless, synthetic BGs demand the introduction of macropores or channels to promote bone augmentation, resulting in the need to use barrier membranes to prevent fibrous tissue penetration. In contrast to general porous synthetic BGs, HC-S has the function of preventing fibrous tissue penetration. Thus, HC-S combines the advantages of synthetic BGs with their ability to prevent fibrous tissue penetration, owing to which, HC-S may be used in the same manner as autologous bone BGs, without concern for complications and unexpected results.

Recent research evaluated the vertical bone augmentation abilities of synthetic BGs and GGs using a similar approach, i.e., vertical augmentation in the rabbit calvarium. Sheikh et al. evaluated the vertical bone augmentation abilities of anabolic conjugate (C3)-containing brushite and monetite dense BGs (a cylinder with a diameter of 9.5 mm and height of 4 mm). The new bone height 12 weeks after implantation of the C3-containing brushite and monetite-dense BGs were 1.8 and 2.7 mm, respectively [61]. Thus, even at 12 weeks post-implantation, the new bone did not form in the upper regions of C3-containing brushite and monetite BG. Furthermore, Kim et al. reported the vertical bone augmentation abilities of calcium sulfate GGs combined with small molecules for promoting bone formation (sodium butyrate and dimethyloxalylglycine) [62]. They filled the GGs in a plastic cylinder: the bottom face of the cylinder was open and the GGs were directly attached to the rabbit calvarium, while the cylinder top face was closed with a plastic lid. The spaces generated between the GGs acted as 3D pores. The new bone heights at 2 and 8 weeks post-implantation were ~1 and ~3 mm, respectively. Furthermore, ~90% of the interior of the plastic cylinder was occupied by tissues, and not bone, even at 8 weeks post-implantation. In the present study, the heights of newly formed bone in the HC-Ss and HC-Rs were 3.9 ± 0.1 and 2.2 ± 0.4 mm at 4 weeks post-implantation and 3.9 ± 0.1 and 3.3 ± 0.4 mm at 12 weeks post-implantation, respectively. These findings indicate that HC scaffolds have higher vertical bone augmentation ability than previously reported scaffolds, including supplements for promoting bone formation. Furthermore, the uniaxial channels of HC scaffolds seem to be more effective for vertical bone augmentation than 3D pores generated between GGs, although the scaffold composition and micro- and nanopores also affect it.

Xu et al., evaluated the in vitro effects of pore shape on cell attachment, proliferation, and ALP activity of osteoblast-like cells (MC3T3) using β-TCP scaffolds with square, triangular, and parallelogram-shaped pores; the pore aperture areas were approximately 0.9, 0.3, and 2.5 mm^2^, respectively [63]. The authors did not report significant differences in cell attachment, proliferation, or ALP activity between these three scaffolds. However, the following tendency was observed: square > triangle > parallelogram for cell attachment and proliferation; square = triangle > parallelogram for ALP activity. These results suggest that pore aperture shape has a stronger impact on cell attachment, proliferation, and ALP activity than pore aperture area, which is consistent with the findings of our study. Furthermore, Shao et al. evaluated in vivo bone formation ability of calcium silicate scaffolds with pores of ~130 and ~320 μm in the Z axial direction; the scaffolds possessed equal-sized pores (~300 μm) in the XY axial direction [64]. Although the scaffold with smaller-sized pores (~130 μm in the Z axial direction) formed a larger volume of bone at 4 weeks post-implantation, the scaffold with larger-sized pores (~320 μm in the Z axial direction) formed a larger volume of bone at 8 and 12 weeks post-implantation. Given the findings of Shao et al. and our present and previous studies [41], pores of approximately 300 μm is appropriate for long-term bone formation.

The results of our study contribute to the development of scaffolds for orthopedic bone reconstruction. Owing to uniaxial channels, HC scaffolds can form new bone, bridging the stumps of separated bones and reconstructing segmental bone defects [37,42]. The reconstruction ability of HC scaffolds is higher than other porous scaffolds [65,66]. Scaffolds cannot always have surface contact with the cross-section surfaces of bone stumps. Thus, a space forms between the scaffold and the bone stump frequently, often promoting fibrous tissue penetration into the scaffolds. The findings of our study suggest that control of the channel or pore aperture characteristics allows the prevention of fibrous tissue penetration in orthopedic bone reconstruction as well as dental bone augmentation.

## 5. Conclusions

We evaluated the effects of channel aperture characteristics on the ingrowths of bone and fibrous tissues by histologically analyzing the vertical bone augmentation in HC-R and HC-S implanted on the rabbit calvarium. The HC-S prevented the penetration of fibrous tissues into the scaffold via the channels; consequently, the new bone became dominant in the space within scaffold channels. In contrast, HC-R could not prevent the fibrous tissue penetration, resulting in lower bone augmentation than HC-S. The collective results from our current and previous studies demonstrate that the longer side length rather than the shorter side length of a rectangular channel aperture profoundly impacts fibrous tissue penetration which affects bone augmentation. Furthermore, scaffolds with different channel aperture shapes provide different results on fibrous tissue penetration and bone augmentation, even though the channel aperture areas of the scaffolds are similar.

## Data Availability

The data presented in this study are available on request from the corresponding author.
